# Combining mesenchymal stem cell sheets with platelet-rich plasma gel/calcium phosphate particles: a novel strategy to promote bone regeneration

**DOI:** 10.1186/s13287-015-0256-1

**Published:** 2015-12-21

**Authors:** Yiying Qi, Lie Niu, Tengfei Zhao, Zhongli Shi, Tuoyu Di, Gang Feng, Junhua Li, Zhongming Huang

**Affiliations:** Department of Orthopedic Surgery, The Second Affiliated Hospital, School of Medicine, Zhejiang University, Hangzhou, 310009 China; Department of Orthopedic Surgery, People’s Hospital of Dongping County, Shandong, China; Department of Orthopedic Surgery, Hangzhou TCM Hospital, Hangzhou, China

**Keywords:** Calcium phosphate particles, Platelet-rich plasma, Mesenchymal stem cell sheet, Bone regeneration, Osteogenic differentiation

## Abstract

**Background:**

Promotion of bone regeneration is important for successful repair of bony defects. This study aimed to investigate whether combining bone marrow-derived mesenchymal stem cell (BMSC) sheets with platelet-rich plasma (PRP) gel/calcium phosphate particles could promote bone formation in the femoral bone defects of rats.

**Methods:**

The proliferation and differentiation of BMSCs or BMSC sheets cultured with calcium phosphate particles and/or PRP were investigated in in vitro. In vivo, 36 2.5 × 5 mm bone defects were randomly divided into groups and treated with either BMSCs/PRP gel, calcium phosphate particles, PRP gel/calcium phosphate particles, a BMSC sheet/calcium phosphate particles, a BMSC sheet/PRP gel/calcium phosphate particles, or were left untreated (n = 6/group). A further 15 bone defects were treated with chloromethyl-benzamidodialkylcarbocyanine (CM-Dil)-labelled BMSC sheet/PRP gel/calcium phosphate particles and observed using a small animal in vivo fluorescence imaging system to trace the implanted BMSCs at 1 day, 3 days, 7 days, 2 weeks, and 4 weeks after surgery.

**Results:**

The expression of collagen type I and osteocalcin genes of BMSCs or BMSC sheets treated with PRP and calcium phosphate particles was significantly higher than that of BMSCs or BMSC sheets treated with calcium phosphate particles or the controls (*P* <0.05). PRP can promote gene expression of collagen III and tenomodulin by BMSCs and in BMSC sheets. The VEGF, collagen I and osteocalcin gene expression levels were higher in the BMSC sheet than in cultured BMSCs (*P* <0.05). Moreover, alizarin red staining quantification, ALP quantification and calcein blue fluorescence showed the osteogenic potential of BMSCs treated with PRP and calcium phosphate particles The implanted BMSCs were detectable at 1 day, 3 days, 7 days, 2 weeks and 4 weeks after surgery by a small animal in vivo fluorescence imaging system and were visualized in the defect zones by confocal microscopy. At 4 weeks after implantation, the defects treated with the BMSC sheet/PRP gel/calcium phosphate particles showed significantly more bone formation than the other five groups.

**Conclusions:**

Incorporation of an BMSC sheet into the PRP gel/calcium phosphate particles greatly promoted bone regeneration. These BMSC sheet and tissue engineering strategies offer therapeutic opportunities for promoting bone defect repair clinically.

## Background

In recent decades, tissue engineering has emerged as an alternative and promising approach for repair of bone defects, using bone marrow-derived mesenchymal stem cells (BMSCs) embedded in a biocompatible scaffold or with growth factors [1]. BMSCs are multipotential cells that can be induced to differentiate into several mesodermal cell types, e.g., osteoblasts, chondrocytes, adipocytes, tenocytes, and myoblasts [2]. In previous studies, BMSCs combined with various materials have been found to regenerate bone defects using cell suspension systems [3–5]. However, the adhesion rate of BMSCs is low due to the low surface-to-volume ratio of scaffolds. The cell suspension systems have fewer advantages or cannot be used with scaffolds of non-porous materials or particles. To address this issue, we adopted a cell transplantation method in which BMSCs are cultured and lifted as a cell sheet structure. The BMSC sheet allows delivery of a much larger number of cells to facilitate tissue regeneration [6, 7]. In addition, the BMSC sheet can be easily detached from the culture substrate, while the extracellular matrix (ECM), the adhesion molecules on the cell surface, and cell-cell interactions remain intact [8].

Among the biomaterials used for bone defect repair, CaP scaffolds have been widely used due to their resemblance to the inorganic composition of bone and have demonstrated excellent biocompatibility and osteoconduction [9–11]. However, it is difficult to use Cap blocks to fill irregularly shaped bone defects [12, 13], and the surgeon must often machine the graft or carve into the surgical site, which increases bone loss, trauma, and surgical time [14]. CaP particles are generally preferred for filling bone defects; however, they are difficult to handle and to maintain in the defects, leading to empty spaces between the particles and bone tissue with mechanical instability [14].

To improve the osteogenic potential of scaffolds, growth factors are usually introduced. Platelet-rich plasma (PRP), which can be easily obtained from blood, is a natural source of growth factors, including platelet-derived growth factor (PDGF), transforming growth factor (TGF-β1, TGF-β2), insulin-like growth factor (IGF-1, IGF-2), and vascular endothelial growth factor (VEGF) [15]. PRP can be activated by thrombin to form PRP gel. In addition, the adhesive properties of PRP gel make possible moulding of CaP particles into even complex bone defects.

The CaP particles and PRP gel were further wrapped by a BMSC sheet, retaining the ECM that is deposited beneath the monolayer during culture. As a potent regulator of cell function and differentiation, the ECM can be well preserved during cell sheet fabrication, thereby enhancing osteoblastic differentiation and improving bone formation. The matrix allows easy re-attachment to CaP particles/PRP gel and additionally provides the stability of, and osteoinduction by, CaP particles. Moreover, the growth factors released from CaP particles/PRP gel can be localised by the BMSC sheet, further promoting bone regeneration.

In this study, we hypothesised that incorporation of a BMSC sheet along with PRP gel/CaP particles would accelerate bone regeneration. We investigated the differentiation of BMSCs or a BMSC sheet cultured with CaP particles and/or PRP in vitro and the ability of PRP gel/CaP particles combined with a BMSC sheet to promote bone regeneration in rat femoral bone defects.

## Methods

### Preparation of CaP particles

**Fig. 1:**
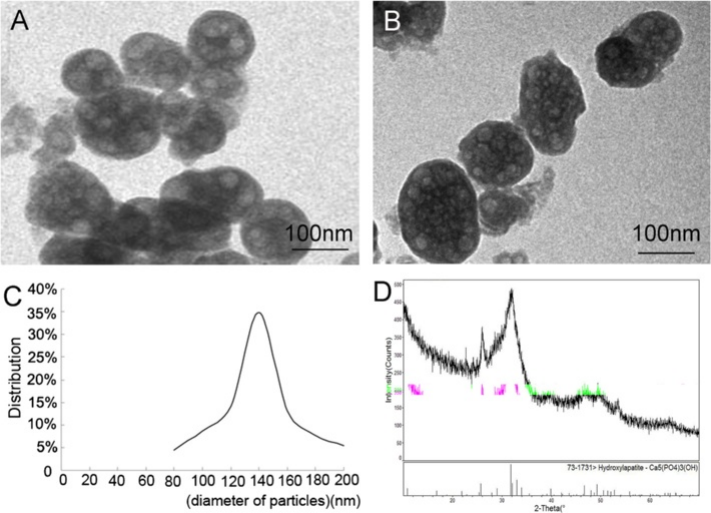
**a** TEM images of CaP particles. **b** TEM of CaP particles after being ultrasonicated by ultrasonic cell crushing apparatus. **c** Particle size distribution of the CaP particles. **d** XRD patterns of CaP particles

CaP particles were fabricated according to the procedures reported by Yurong Cai et al. [16]. Briefly, CaP particles were synthesised by dropwise addition of 5.0 mM CaCl_2_ (60 ml) to 240 mL of solutions containing 1.25 mM Na_2_HPO_4_ and 24 × 10^-4^ M hexadecyl (cetyl) trimethyl ammonium bromide (CTAB) in magnetically stirred vessels at 20 °C. The pH of the solutions was maintained at 9.5 ± 0.5 by the addition of 0.1 M ammonia. The suspension was then stirred at 20 °C for another 24 h to allow completion of particle formation. The precipitate was washed with distilled water. After drying in a vacuum at 40 °C, solids were washed with ethanol to dissolve residual CTAB molecules and separated from the CTAB by five 10-minute cycles of centrifugation at 1,800 g. The CaP particles in the precipitate were then dried in a vacuum at room temperature. Transmission electron microscopy (TEM, JEM-200CX, Tokyo, Japan, operated at 160 KV) (Fig. 1a) showed that the CaP particles were sphere-like with approximate diameters of 140 nm. Figure 1b shows TEM images of CaP particles after being ultrasonicated using an ultrasonic cell crushing apparatus. The size distributions of the nanoparticles were relatively narrow and the average diameter was 140 ± 24 nm (analysed by Image-Pro Plus 6.0, Media Cybernetics, Yena, Germany.) (Fig. 1c). The energy dispersive spectrometer (EDS) analysis showed that the Ca:P ratios of the particles was 1.67 ± 0.02. The x-ray diffraction (XRD, Rigaku D, Tokyo, Japan/max-2550 pc, scan step of 0.02 from 10 ° to 60 °) analysis indicated that the particles were poorly crystalline hydroxyapatite (HA) (Fig. 1d).

### Preparation of PRP and PRP gel/CaP particles composite

Five Sprague-Dawley rats were used to prepare the PRP. The Institutional Animal Care and Use Committee of Zhejiang University approved all animal experimental protocols. Approximately 8 mL of venous blood from the right atrium of each rat were drawn into a sterile tube containing sodium citrate as an anticoagulant for processing PRP. The platelets were enriched by a two-step centrifugation process. During the first centrifugation at 200 × g for 10 minutes in a refrigerated centrifuge 5415R (Eppendorf Corporation, Eppendorf, Germany), the blood components were separated into two phases: one phase comprising PRP and the other comprising erythrocytes and leukocytes. The samples of PRP underwent a second centrifugation at 560 × g for 15 minutes that allowed precipitation of the platelets, which were then re-suspended in 20 μL of plasma. Samples of PRP and whole blood were analysed using an automatic counter (Sysmex F-820, Sysmex Corporation, Tokyo, Japan ). The average platelet concentration in whole blood was 2.36 ± 0.52 × 10^8^/mL, while platelets reached an average concentration of 13.2 ± 1.18 × 10^8^/mL in a processed PRP, which was six-fold the serum level.

**Fig. 2:**
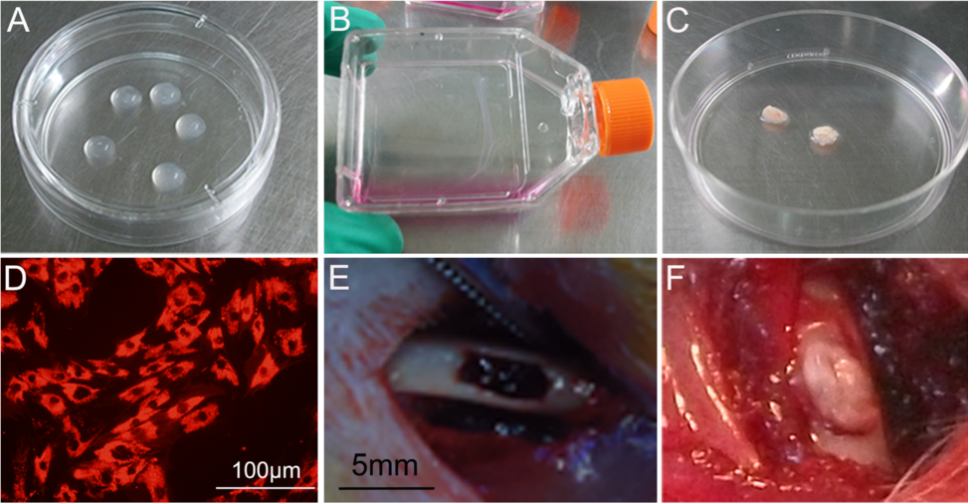
**a** PRP was activated to form PRP gel. **b**, **a** BMSC sheet was harvested using a cell scraper. **c** The PRP gel/CaP particles composite was wrapped by BMSC sheet. **d** BMSCs were stained by CM-Dil. **e** Bone defects were prepared in the cortical bone of femurs. **f** BMSC sheet/PRP gel/CaP particle composite was implanted into bone defects

To prepare the PRP gel, the platelets were activated with thrombin (Sigma, St. Louis, MO USA 5 U/mL in 40 mM CaCl_2_): 10 μL of PRP and 10 μL of thrombin was mixed and incubated at 37 °C for 30 minutes to form PRP gel (Fig. 2a). The PRP gel/CaP particle composite was prepared by simultaneously mixing 10 μL of PRP and 10 μL of thrombin with 10 mg of particles. After mixing, the composite scaffolds were incubated at 37 °C for 30 minutes to form a strong PRP gel/CaP particle composite [17, 18]. Then the PRP gel and PRP gel/CaP particle composites were observed by scanning electron microscopy (SEM).

### BMSC culture, cell sheet preparation and fabrication of cell sheet-scaffold constructs

Rat BMSCs were isolated from the bone marrow of young adult male Sprague-Dawley rats following the procedure described by Nakamura et al. [8] and Kaur et al. [19]. Briefly, rats were euthanised with CO_2_, and the femurs and tibias were then removed. The bones were washed in α-minimal essential medium (α-MEM) supplemented with 1 % (v/v) penicillin/streptomycin (Gibco, Thermo Fisher Scientific Inc. New York, USA ). Both ends of the femurs and tibias were cut away from the epiphysis, and the bone marrow was flushed out of the bone with 10 mL of medium in a syringe. The cells were filtered through a 70-μm cell strainer and centrifuged at 300 g for 5 minutes. The cell pellet was re-suspended in 10 mL of α-MEM supplemented with 10 % foetal bovine serum (FBS, Gibco, Thermo Fisher Scientific Inc. New York, USA) and plated in a culture plate. Cells were maintained at 37 °C in a humidified atmosphere with 5 % CO_2_, and the medium was changed every 2 days. When adherent cells reached 80–90 % confluence, they were detached with 0.25 % trypsin-EDTA Gibco, Thermo Fisher Scientific Inc. New York, USA) and replanted at 1:3 in regular growth medium to allow for continued passaging. BMSCs at passages 3–4 were used in the experiments. Monolayer cultured cells were detached by mechanical scratching and filtered through a stainless-steel mesh filter to eliminate cell aggregates from the single-cell suspension. After centrifugation, cells were blocked with 1 % bovine serum albumin for 15 minutes. Cells were then incubated with 5 μL of antibodies (APC-CD44, APC/Cy7-CD45, PE/Cy7-CD73, Percp/Cy5.5-CD90 and FITC-CD105) (Biolegend, San Diego, CA, USA) for 30 minutes on ice. Appropriate isotype control antibodies were used to exclude non-specific binding. After washing, the samples were analysed using a BD FACSCalibur (BD Biosciences, San Jose, California, USA).

To create the cell sheet, the released cells were seeded at 4 × 10^4^ cells/cm^2^ into flasks cultured in 10 mL of α-MEM supplemented with 10 % FBS (Gibco, Thermo Fisher Scientific Inc. New York, USA) for at least 1 week. Cells were maintained at 37 °C in a humidified atmosphere with 5 % CO_2_, and the medium was replaced daily. After 1 week, the layered BMSCs were formed. The cells were then rinsed with phosphate-buffered saline (PBS; Gibco, Thermo Fisher Scientific Inc. New York, USA) twice, and then lifted as a cell sheet using a scraper (Fig. 2b). The intact BMSC sheet was then wrapped around the PRP gel/CaP particles or single CaP particles (10 mg) for in vivo implantation (Fig. 2c).

To track the implanted BMSC sheet, first the BMSCs were stained with chloromethyl-benzamidodialkylcarbocyanine (CM-Dil, Molecular Probes, Thermo Fisher Scientific Inc. New York, USA ) following the method described by Kruyt et al. [20] (Fig. 2d). BMSCs in suspension were washed with PBS and incubated with CM-Dil at a concentration of 2 μg/mL PBS for 5 minutes at 37 °C and 15 minutes at 4 °C. After two washings in PBS, the cells were re-suspended in α-MEM and cultured to form a BMSC sheet. This fluorescent marker intercalates within the cell membrane lipid bilayer and is useful for tracking live cells in vivo [21, 22]. The dye does not influence cell metabolism or viability [23].

### Proliferation of BMSCs stimulated by CaP particles

Based on the particle weight to medium volume ratio, various concentrations of particles were prepared. Concentrations of 0.001, 0.01, and 0.1 % of particles corresponded to 0.01, 0.1, and 1.0 mg of particles per mL of medium. The particle solutions were ultrasonicated using ultrasonic cell crushing apparatus (SL-1000D, Nanjing Shunliu Instrument Co. Ltd., Nanjing, China) at a frequency of 20 kHz and maximum power (1000 kW) for 10 minutes in a sealed sterile container to minimise agglomeration of the particles before adding them to the cell culture. The proliferation of BMSCs plated in 96-well plates (2 × 10^3^ cells per well) co-cultured without (determining the positive control) or with CaP particles at concentrations of 0.001, 0.01, and 0.1 % was evaluated using a 3(4,5 dimethylthiazol)-2,5 diphenyltetra-zolium (MTT) assay. The cell-free material present served as blank samples. After culturing for 1, 3, or 7 days, the MTT assay was performed according to the cell proliferation kit protocol (Sigma, St. Louis, MO USA). This assay was repeated three times per group.

### Differentiation of BMSCs cultured with CaP particles and/or PRP in vitro

The differentiation of BMSCs or a BMSC sheet cultured without (the control group) or with CaP particles (0.001 %) and/or PRP for 7 days was examined by evaluating the expression levels of the genes encoding collagen type I, osteocalcin, collagen III, tenomodulin and VEGF. To assess osteogenic differentiation, BMSCs were seeded in a six-well plate at a density of 2 × 10^5^ per well; each BMSC sheet was cultured in osteogenic medium consisting of α-MEM supplemented with 10 mM Na-β glycerophosphate, 0.2 mM ascorbic acid, and 10^-8^ M dexamethasone (Sigma, St. Louis, MO USA).

For non-osteogenic differentiation, BMSCs were seeded in a six-well plate at a density of 2 × 10^5^ per well; the BMSC sheet was then cultured in α-MEM. Particle suspensions or PRP (10 μL) with particle suspensions were added to cells to a particle concentration of approximately 0.01 mg/mL (0.001 %). As controls, cells were subjected to the same manipulations but incubated in the absence of particles or PRP.

Moreover, after culturing without or with CaP particles (0.001 %) and/or PRP for 14 days, the osteogenic differentiation of the BMSCs was examined by means of Alizarin red staining quantification and alkaline phosphatase (ALP) quantification. The ALP activity of BMSCs was determined using a pNPP Phosphatase Assay kit (Nanjing Jiancheng Bio-engineering Co., Ltd. Nanjing, China). ALP activity was normalised to the total protein content determined using a bicinchoninic acid (BCA) assay. For Alizarin red staining quantification, the Alizarin red stain was dissolved in 500 μL of 5 % SDS/HCl (m/v, HCl = 0.5 M) solution. Half an hour later, the absorbance of the solution at 405 nm was determined using a microplate reader. A Cell Counting Kit-8 (CCK-8) was used to assess BMSCs proliferation after culturing without or with CaP particles (0.001 %) and/or PRP for 14 days. The quantification of Alizarin red staining per cell was determined by dividing the optical density (OD) value of Alizarin red by that of CCK-8 staining.

Calcein blue (C15H15NO7) has been administered by intraperitoneal injection to label newly calcified tissues in vivo. In the present study, we used calcein blue to label newly mineralised nodules in vitro. Calcein blue powder (Sigma, St. Louis, MO USA) was dissolved in 100 mM KOH (diluted in distilled water) and filtered to create the 30 mM stock solution. Following culture of BMSCs without or with CaP particles (0.001 %) and/or PRP for 14 days, 30 μM (the final concentration in culture medium) calcein blue was added to the medium and incubated overnight. Prior to microscopic examination and photography, cultures received fresh medium without fluorochrome to prevent generation of a non-specific fluorescent background. Calcein blue emits blue fluorescence that can be detected using a Sapphire green fluorescent protein (GFP) filter.

### Quantitative reverse transcription-polymerase chain reaction (qRT-PCR) assays for cultured BMSCs, in vitro fabricated BMSC sheets, and particles and/or PRP-induced BMSCs

Total cellular RNA was extracted from cells with indicated treatments using TRIzol reagent (Invitrogen Thermo Fisher Scientific Inc. New York, USA ). Complementary DNAs (cDNAs) were synthesised using a commercial kit (SuperScript II Reverse Transcriptase, Invitrogen, Thermo Fisher Scientific Inc. New York, USA ) according to the manufacturer’s recommendations. Each group involved four wells. Real-time polymerase chain reaction (PCR) quantification was performed using the SYBR Premix Ex Taq Kit (TaKaRa, Tokyo, Japan) on an iQTM5 multiplex real-time fluorescence quantitative PCR instrument (Bio-Rad, California, USA). The gene expression levels of osteocalcin (OCN), collagen I, collagen III, tenomodulin, and VEGF were determined, following the procedures described by Nakamura et al. [8] and Kaur et al. [19]. Thermal cycle conditions comprised 1 minute at 95 °C for activation of the Universal mixture AmpliTaq Gold Polymerase, followed by 45 cycles of 10 s at 95 °C for denaturing and 25 s at 62 °C for annealing and extension. Primers for target genes and the internal control gene are listed in Table 1. 18S rRNA was used as an internal control to adjust for differences between samples.

**Table 1:** Nucleotide primers used for qRT-PCR

Genes	Oligonucleotide sequence(5′-3′)	Product size (bp)
18S rRNA	Forward: GAATTCCCAGTAAGTGCGGGTCATA	105
	Reverse: CGAGGGCCTCACTAAACCATC	
Collagen type I	Forward: TGGCCAAGAAGACATCCCTGAAGT	81
	Reverse: ACATCAGGTTTCCACGTCTCACCA	
Osteocalcin	Forward: CTCACTCTGCTGGCCCTGAC	111
	Reverse: CACCTTACTGCCCTCCTGCTTG	
Collagen III	Forward: GTGTCTGCGACTCGGGATCT	120
	Reverse: TAGAAGGCTGTGGACATATTGCA	
Tenomodulin	Forward: GGACTTTGAGGAGGATGGTGAA	83
	Reverse: GGACCACCCATTGCTCAT TC	
Vascular endothelial growth factor	Forward: GTCACCACCACACCACCATCGT	76
	Reverse: CTCCTCTCCCTTCATGTCAGGCT	

### Animals and surgical procedure

Eighteen 12-week-old male Sprague-Dawley rats (weighing about 300–400 g each) were used in this study. The Institutional Animal Care and Use Committee of Zhejiang University approved all animal experimental protocols. According to the procedures of a previous study [24], under anaesthesia induced with an intraperitoneal injection of 8 % chloral hydrate (400 mg/kg body weight), femoral bones were carefully exposed by exfoliation. After the periosteum was dissected, artificial bone cavities with 2.5 and 5.0 mm transverse and longitudinal extensions were prepared in the cortical bone of both femurs by drilling with a round bur attached to a dental hand piece (Fig. 2e). The bone cavity was carefully and completely washed prior to insertion of bone marrow cells.

Thirty-six bone defects were randomly divided into six groups (n = 6/group). The bone defects were either untreated or treated with BMSCs/PRP gel, CaP particles (10 mg), PRP gel/CaP particles, BMSC sheet/CaP particles, or a BMSC sheet/PRP gel together with CaP particles (Fig. 2f). The rats were euthanized 4 weeks after surgery. Fifteen additional mice with bone defects implanted with the CM-Dil-labelled BMSC sheet/PRP gel/CaP particles were sacrificed at 1 day, 3 days, 7 days, 2 weeks and 4 weeks after surgery. The femurs were immediately dissected from the rats at 1 day, 3 days, 7 days, 2 weeks and 4 weeks after surgery, and the bone samples were examined using a small animal in vivo fluorescence imaging system (Maestro, MA, USA). For confocal laser scanning microscopy (CLSM, LSM 510, Zeiss, Yena, Germany) observation, the samples were fixed in neutral buffered formalin, decalcified and placed into a 0.1-mol/L phosphate buffer solution containing 30 % sucrose for 48 h at 4 °C and embedded into optimum cutting temperature compound (OTC). Sagittal plane sections (4 μm) were cut using a frozen sectioning machine (Lecia, Wetzlar, Germany). The images were obtained after staining of the sections with Hoechst 33258 (Sigma, St. Louis, MO USA).

### Gross observation and radiological examination

Postoperative activities, food intake, and wound healing were evaluated in the animals. The animals were euthanized, after which bone repair and callus growth were evaluated using samples collected through the original incision. Anterior–posterior radiographs of the bilateral femur were obtained to assess bone healing at 4 weeks after implantation.

### Histological examination and histomorphometric analysis

Specimens from the bone defect sites were fixed in 10 % paraformaldehyde (Shanghai Biological Engineering Co., Ltd., Shanghai, China), decalcified with formate-sodium formate, and embedded in paraffin. Sagittal plane sections (7 μm) from each defect were prepared, stained with haematoxylin and eosin, and examined under a light microscope (Olympus, Tokyo, Japan). For histomorphometric analysis, five sequential sections per sample were selected for evaluation under low magnification, allowing coverage of the entire defect. Using the image analytical software Image-Pro Plus, all slides were examined by two independent observers to identify the type of bone tissue. The extent of bone formation was indicated by the percentage of bone tissue area within the cortical bone defect site, and an average value was calculated for each sample.

### Statistical analysis

Results were analysed by one-way analysis of variance and are expressed as means and standard deviations. Data analyses were performed using the SPSS software (ver. 15.0; SPSS, Inc., Chicago, IL, USA). Tukey’s test was used for multiple comparisons, and the level of significance was set at *P* <0.05.

## Results

### Flow cytometry

**Fig. 3:**
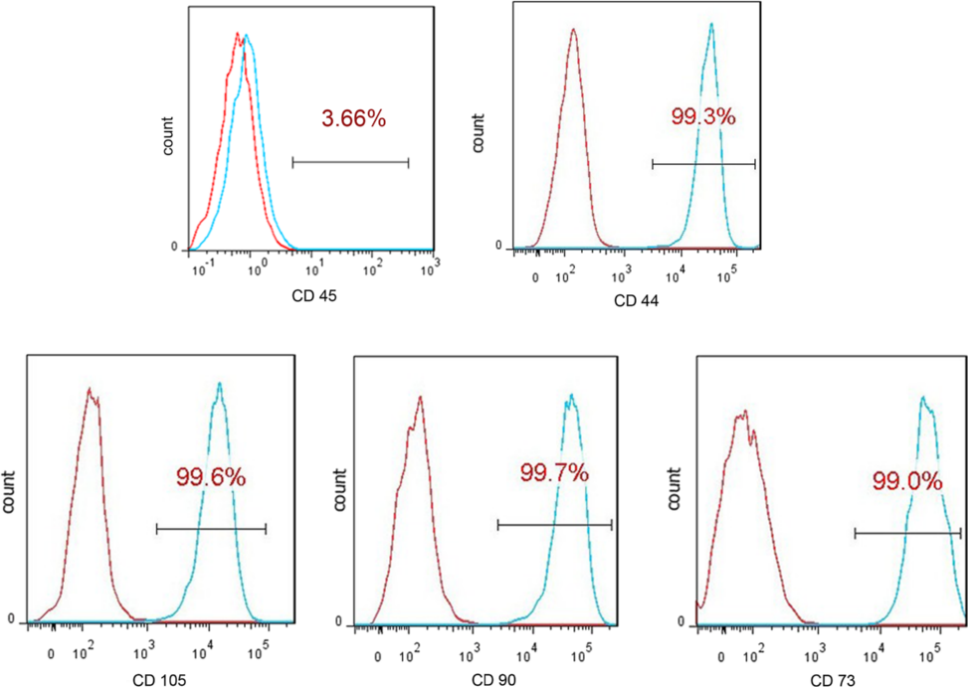
Flow cytometry results for BMSCs, expressing CD44, CD73, CD90 and CD105, but not CD45. *Red histogram* is the negative control. *Left side* of the *blue histogram* (in the negative control part) represents the negative expression of CD marker; *right side* represents the positive expression of CD marker

On flow cytometry results the cultured cells expressed CD44, CD73, CD90, and CD105 but not CD45, which is characteristic of BMSCs (Fig. 3).

### SEM observation

**Fig. 4:**
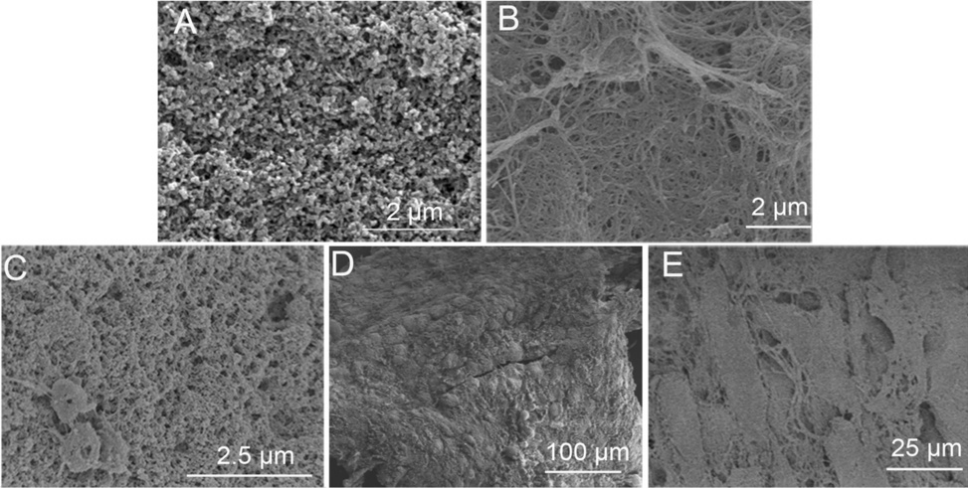
Scanning electron microscopy of CaP particles (**a**), PRP gel (**b**), PRP gel/CaP particles (**c**), BMSC sheet-wrapped CaP particles (**d**) and BMSC sheet-wrapped PRP gel/CaP particles composite (**e**)

After the CaP particles were fabricated, they were observed by SEM. Figure 4a shows the round shape of the CaP particles, which tended to agglomerate. After the PRP gel and PRP gel/CaP particles were incubated at 37 °C for 30 minutes, and lyophilized in a freeze dryer (Heto Power Dry LL1500), the PRP gel and PRP gel/CaP particle composites were observed by SEM (Fig. 4b, c). The polymerisation of the PRP gel formed a complex network of fibres (Fig. 4b). The PRP fibres partially covered the surface and penetrated the microstructure of the CaP particles; the particles were connected to each other through the PRP fibres (Fig. 4c). After the PRP gel/ CaP particles formed, then the intact BMSC sheet was then wrapped around the PRP gel/CaP particles or single CaP particles and the composites were incubated at 37 °C in a humidified atmosphere with 5 % CO_2_ for 2 h. After fixation and dehydration, the composites were observed by SEM. The BMSC sheet wrapped around the particles and the shape of the cells could be seen (Fig. 4d). The BMSC sheet completely wrapped the surface of the PRP gel/CaP particle composite (Fig. 4e). The cells juxtaposed tightly together and formed dense cell layers.

### BMSC proliferation

**Fig. 5:**
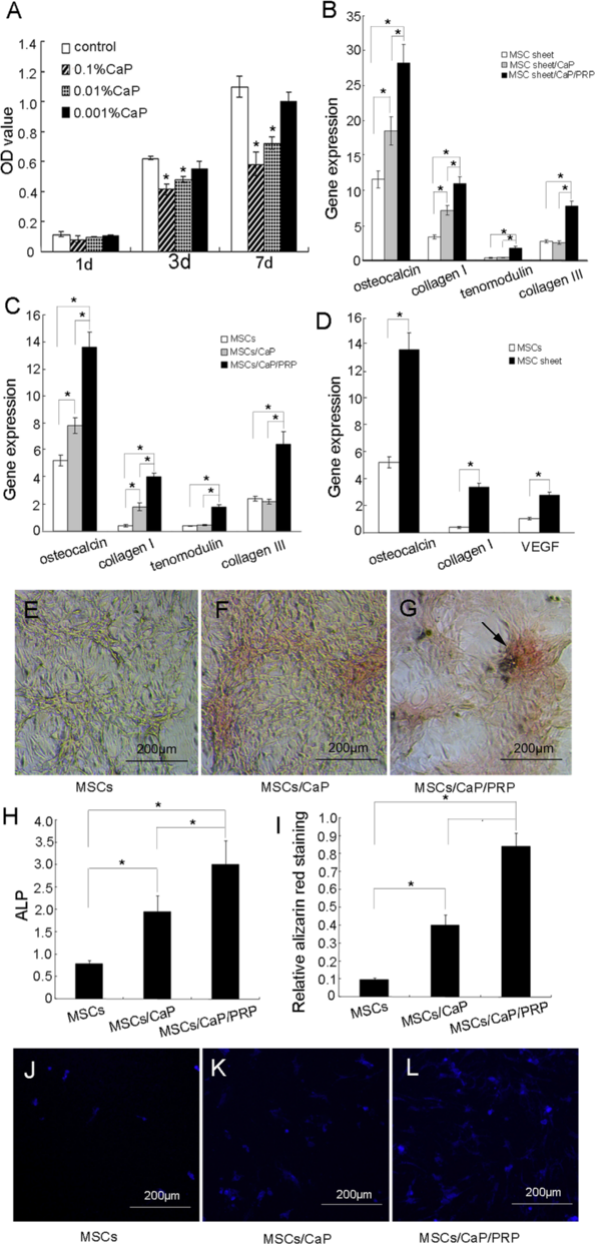
**a** Dose effects of CaP particles on BMSC proliferation after culturing for 1, 3 and 7 days (mean ± SD) (*P <0.05). **b** Gene expressions of BMSC sheet cultured with CaP particles or/and *PRP* for 7 days (*P <0.05). **c** Gene expressions of BMSCs cultured with CaP particles or/and PRP for 7 days (*P <0.05). **d** Osteocalcin, collagen I and vascular epithelial growth factor (VEGF) gene expression of the BMSC sheet and cultured BMSCs (*P <0.05). **e**, **f**, **g** The alizarin red staining of BMSCs cultured with PRP/CaP particles, CaP particles or without treatment for 14 days. **e** The alizarin red staining in the controls was much weaker. **f** The alizarin red staining was increased when treated with CaP particles. **g** A calcification node formed in the BMSCs co-cultured with CaP particles/PRP. **h** ALP activity of BMSCs cultured with PRP/CaP particles, CaP particles or without treatment for 14 days; ALP activity was 3.01 ± 0.51 nmol/s/mg protein, 1.95 ± 0.36 nmol/s/mg protein and 0.79 ± 0.08 nmol/s/mg protein in the CaP particles/PRP group, CaP particles group and control group respectively. **i**: Quantification of alizarin red staining of BMSCs cultured with PRP/CaP particles, calcium phosphate particles or without treatment for 14 days; expression of BMSCs co-cultured with CaP particles/PRP was 0.84 ± 0.07, the cells with CaP particles was 0.4 ± 0.06 and the control was 0.09 ± 0.01. **j**, **k**, **l** Calcein blue staining of BMSCs without treatment (**j**) or cultured with CaP particles (**k**) or PRP/CaP particles (**l**) for 14 days

A decrease in BMSC proliferation for particle concentrations greater than 0.001 % was observed compared to the control (*P* <0.05) (Fig. 5a). There was a dose-dependent effect; i.e., the highest concentration resulted in the greatest inhibition of BMSC proliferation.

### Analysis of gene expression

After BMSCs or a BMSC sheet cultured without or with CaP particles (0.001 %) and/or PRP for 7 days, gene expression was detected. The expression of the collagen type I and osteocalcin genes in BMSCs and the BMSC sheet treated with CaP particles was notably upregulated compared to that of the controls (*P* <0.05). The expression levels of the collagen type I and osteocalcin genes of BMSCs alone and BMSCs treated with CaP particles and PRP were significantly higher than those of CaP particles and the control group (*P* <0.01) (Fig. 5b, c). In addition, PRP promoted collagen III and tenomodulin gene expression in BMSCs and the BMSC sheet (Fig. 5b, c). The BMSC sheet was associated with greater expression of the VEGF, collagen I, and osteocalcin genes than were cultured BMSCs (*P* <0.05) (Fig. 5d).

### Alizarin red staining, ALP and alizarin red staining quantification

The alizarin red staining was increased in BMSCs co-cultured with CaP particles/PRP compared to CaP particles or controls (Fig. 5e-g). A calcification node formed, indicating that the CaP particles and PRP synergistically induced osteogenic differentiation of BMSCs. The alizarin red staining in the controls was considerably weaker.

At 14 days, the ALP activity was 3.01 ± 0.51 nmol/s/mg protein in cells co-cultured with CaP particles/PRP, which was significantly higher than 1.95 ± 0.36 nmol/s/mg protein for cells co-cultured with CaP particles and 0.79 ± 0.08 nmol/s/mg protein for BMSCs in the control group (*P* <0.05) (Fig. 5h).

Alizarin red staining quantification also showed that BMSCs co-cultured with CaP particles/PRP had the highest expression (0.84 ± 0.07), significantly higher than that of the cells combined with CaP particles (0.4 ± 0.06) and the controls (0.09 ± 0.01) (*P* <0.05) (Fig. 5i).

On calcein blue stained images there was little fluorescence in the control group (Fig. 5j). The formation of mineralised nodules marked by calcein blue was clearly demonstrated by an increase in both the area and intensity in BMSCs co-cultured with CaP particles or with PRP (Fig. 5k, l). The mineralised nodule staining pattern of calcein blue fluorescent images was in agreement with the Alizarin red staining pattern.

### Tracking of implanted BMSC sheet/PRP gel/CaP particles

**Fig. 6:**
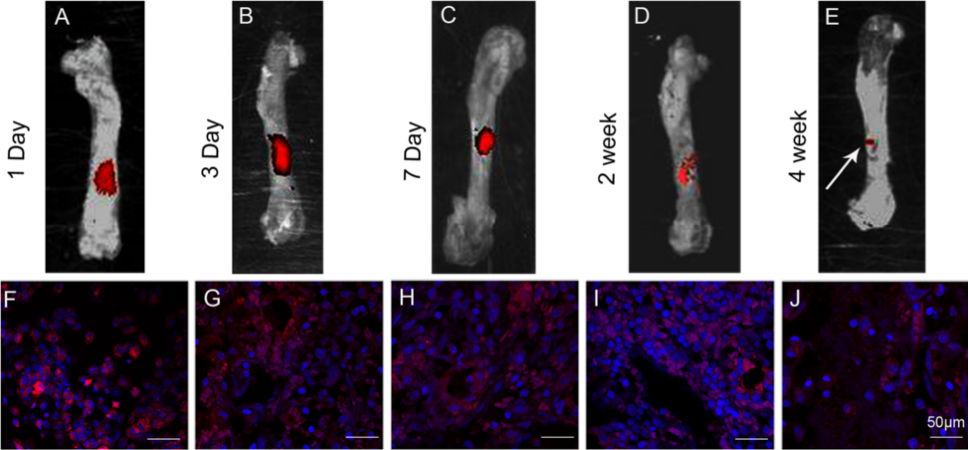
Fluorescence images obtained by a small animal in vivo fluorescence imaging system at 1 day (**a**), 3 days (**b**), 7 days (**c**), 2 weeks (**d**), 4 weeks (**e**) after the bone defects were treated with CM-Dil-labelled BMSC sheet/PRP gel/CaP particles. Red fluorescence in the bone defect zones was visualised using a small animal in vivo fluorescence imaging system, which confirmed the presence of the implanted BMSCs. After further frozen sectioning, red fluorescence was also visible in the defect zones by confocal microscopy at 1 day (**f**), 3 days (**g**), 7 days (**h**), 2 weeks (**i**) and 4 weeks (**j**). However, the fluorescence intensity gradually weakened. The *red* and the *blue* colours were stained by CM-Dil and Hoechst 33258, respectively

Red fluorescence in the bone defect zones was visualised using a small animal in vivo fluorescence imaging system after implantation at 1 day, 3 days, 7 days, 2 weeks, and 4 weeks, which confirmed the presence of the implanted BMSCs (Fig. 6a–e). After frozen sectioning, red fluorescence was also visible in the defect zones by confocal microscopy (Fig. 6f–j). However, the fluorescence intensity gradually weakened.

### Gross observation

**Fig. 7:**
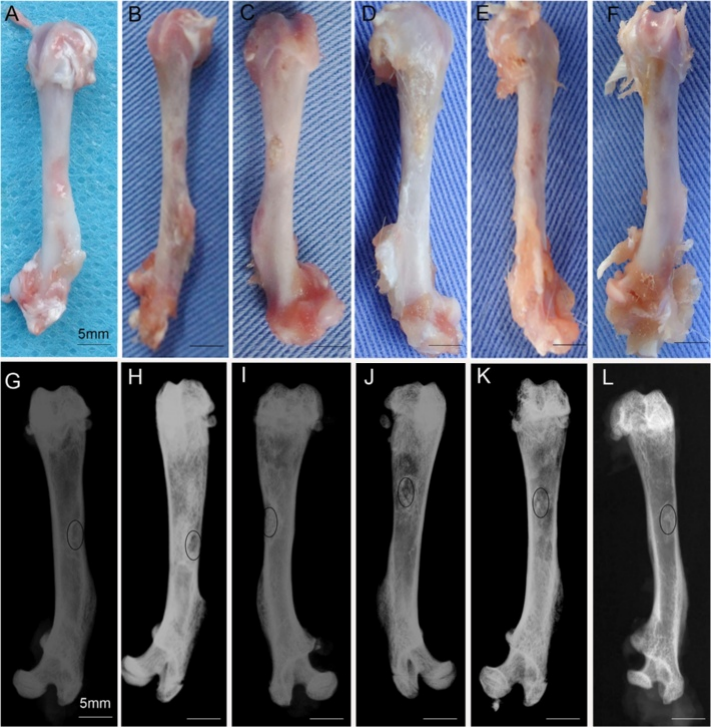
Gross observation and radiographic analysis of repaired bone defects at 4 weeks after surgery. **a** and **g** Control group. **b** and **h** BMSCs/PRP gel group. **c** and **i** CaP particles group. **d** and **j** PRP gel/CaP particles group. **e** and **k** BMSC sheet/CaP particles group. **f** and **l** BMSC sheet/PRP gel/CaP particles group

All rats ate a normal diet and behaved appropriately after surgery, and all survived until euthanasia with no apparent complications, such as incision infection or skin necrosis. At 4 weeks, in the control group, the bone defect sagged inwards and a thin layer of bone covered the defects, which distinguished them from normal bone tissue (Fig. 7a). In the BMSCs/PRP group, the bone defect was covered by a thin layer of bone, which was also distinguished from normal bone tissue (Fig. 7b). A large part of the defect in the CaP particle group was filled with CaP particles and could be recognised from the surface (Fig. 7c). In the CaP particles/PRP gel group, the surface of the defects was filled with thin bone tissue and few particles (Fig. 7d). In the BMSC sheet/particles group and the BMSC sheet/particles/PRP gel group, bone defects were regenerated with dense bone tissue (Fig. 7e, f). However, in the BMSC sheet/particle group, the bone tissue in the middle of the bone defect was much thinner. The defects in the BMSC sheet/particles/PRP gel group were indistinguishable from normal bone tissue (Fig. 7f).

### Radiographic examination

X-ray images were obtained at 4 weeks after surgery to evaluate bone healing within defects. Small portions of the bone defect had not regenerated in the control group (Fig. 7g). Thin bone tissue was regenerated in the BMSCs/PRP group (Fig. 7h). In the particles group, the defects were mostly filled with non-degraded particles (Fig. 7i). Bone tissue was regenerated in the CaP particles/PRP gel group and the BMSC sheet/CaP particle group (Fig. 7j, k). The bone tissue in the CaP particles/PRP gel group was much thinner than that of the BMSC sheet/CaP particles group. Finally, the cortical bone tissue had completely regenerated in the BMSC sheet/PRP gel/CaP particles group (Fig. 7l).

### Histological analysis

**Fig. 8:**
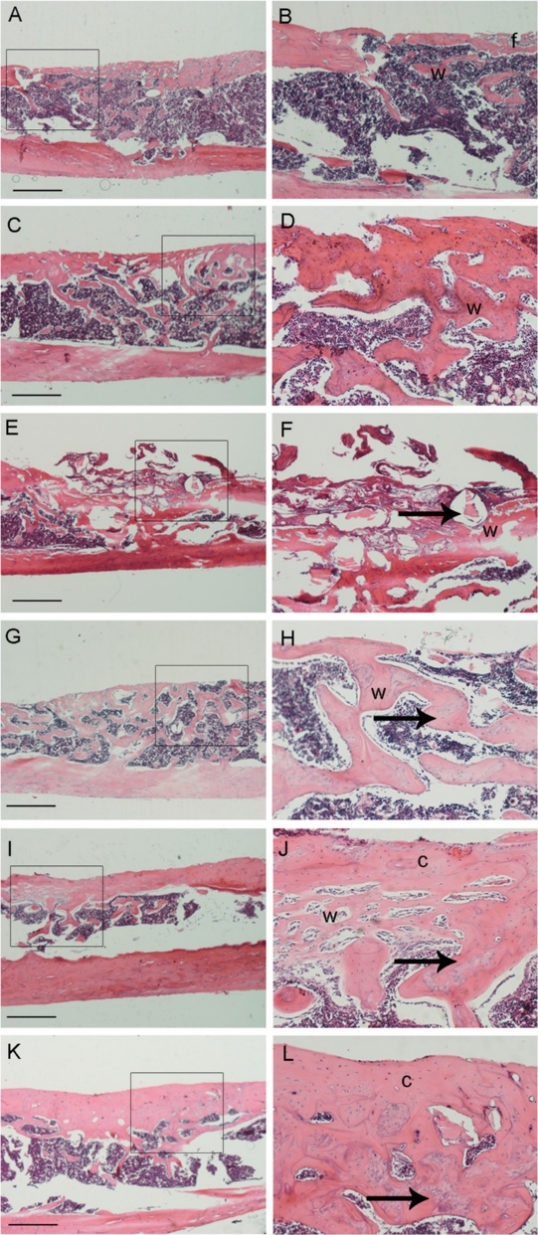
Histological examination of the repaired bone tissues at 4 weeks after implantation. **a** and **b** Control group. **c** and **d** BMSCs/PRP gel group. **e** and **f** CaP particles group. **g** and **h** PRP gel/CaP particles group. **I** and **j** BMSC sheet/CaP particles group. **k** and **l** BMSC sheet/PRP gel/CaP particles group. **b**, **d**, **f**, **h**, **j**, **l** higher magnifications of **a**, **c**, **e**, **g**, **i**, **k** respectively. *w* woven bone tissue, *c* cortical bone tissue, *f* fibrous tissue. *Arrow* residual particles. *Scale bar* 300 μm

In the control group, much thinner woven bone tissue bridged the bone defects. However, some fibrous tissue had filled the surface of the regenerated bone tissue (Fig. 8a, b). In the BMSCs/PRP gel group, a quantity of woven bone filled the bone defects and the bone tissue was much thinner in the middle of the defects (Fig. 8c, d). In the CaP particles group, woven bone had formed at the edge of the defects, whereas other parts of the defects were filled with fibrous tissue and CaP particles. Some particles were surrounded by fibrous tissues and were outside the defects, due to the instability of the particles (Fig. 8e, f). In the CaP particles/PRP gel group, some woven bone filled the bone defects (Fig. 8g, h). In the BMSC sheet/CaP particles group, the defects were regenerated with cortical bone tissue, which was much thinner than normal cortical bone, especially in the middle of the defect (Fig. 8i, j). In the BMSC sheet/PRP gel/CaP particle group, the defects were filled with well-distributed and thick cortical bone tissue (Fig. 8k, l). The shadow of particles was obvious and the particles were regenerated with new bone tissue.

### Histomorphometric analysis

**Fig. 9:**
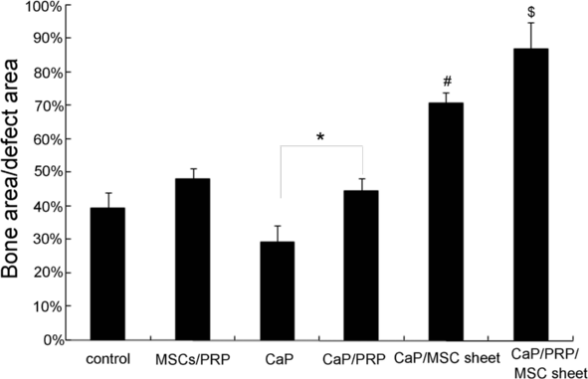
Extent of bone formation was expressed as a percentage of bone tissue area within the original cortical bone defect area. *Error bars* represent means ± SD (n = 5); *P <0.05; ^#^CaP/BMSC sheet vs CaP/*PRP*, CaP, BMSCs/PRP, Control, *P* <0.05; ^$^CaP/PRP/BMSC sheet vs other five groups, *P* <0.05

At 4 weeks, the area of regenerated bone tissue in the BMSC sheet/PRP gel/CaP particle group was significantly greater (87 ± 8.2 %) than that in the other five groups (*P* <0.05) (Fig. 9). In addition, the area of regenerated bone in the BMSC sheet/CaP particle group (71 ± 3.6 %) was significantly greater than that of the control group (39 ± 4.7 %), the BMSCs/PRP group (48 ± 3.4 %), the CaP particle group (29 ± 5.1 %), and the CaP particles/PRP gel group (45 ± 3.8 %) (*P* <0.05). No significant difference in the area of bone tissue was observed among the control group, the CaP/PRP gel group, and the BMSCs/PRP group; all had greater values than did the CaP particle group.

## Discussion

The results of this study demonstrated that incorporation of a BMSC sheet along with PRP gel/CaP particles greatly promoted bone regeneration. The BMSC sheet and PRP gel can provide cells and growth factors to accelerate bone formation. Moreover, we revealed that the BMSC sheet/CaP particles also had a significant positive effect on bone formation, compared to CaP particles/PRP gel and CaP particles.

This in vitro study demonstrated that CaP particles have dose-dependent effects on BMSC proliferation. BMSC proliferation was decreased by addition of 0.1 % and 0.01 % CaP particles compared to the control group, which was consistent with the results of a previous study [25]. Based on the cell proliferation assay, the appropriate concentration of CaP particles (0.001 %) was then used to evaluate their effect on induction of osteogenic differentiation of BMSCs and a BMSC sheet. BMSCs and BMSC sheets co-cultured with CaP particles (0.001 %) highly expressed osteocalcin and collagen type I genes, and exhibited positive alizarin red staining and ALP activity, which indicated osteogenic differentiation. Moreover, CaP particles combined with PRP exerted a synergistic effect on the osteogenic differentiation of BMSCs and BMSC sheets. The osteocalcin and collagen type I genes of BMSCs and BMSC sheets were highly upregulated, alizarin red staining was highly positive, and the alizarin red staining intensity and ALP activity were highest. PRP contains several growth factors, including PDGF, TGF-β1, and VEGF, which can induce osteogenic differentiation of BMSCs. Mouse BMSCs treated with PRP had greater ALP activity and collagen type I gene expression [26]. However, the specific growth factors in PRP were not quantified in this study. A previous study reported that the levels of growth factors (PDGF, TGF-β1 and VEGF) in PRP samples from goats were significantly higher than those of circulating plasma and the osteogenic differentiation of BMSCs was promoted by PRP [27]. Zhou et al. [28] reported that the addition of PRP had a synergistic effect on osteogenic differentiation of BMSCs, which was consistent with our results.

Moreover, previous studies also showed that PRP induces tenogenic differentiation of adult stem cells in vitro [29–31] and PRP combined with adult stem cells promote tendon repair in vivo [32, 33]. PRP can promote tenogenic differentiation and increase expression of such tenocyte-related genes as collagen type I, collagen type III, tenomodulin [29–31], in agreement with our results. As shown in the study, PRP promoted collagen III and tenomodulin gene expression by BMSCs and BMSC sheets in vitro.

Basic research has demonstrated the ability of PRP to promote bone healing [34, 35]. In this study, the PRP gel/CaP particle group and the PRP gel/BMSCs group had greater bone formation than did the CaP particle group. CaP particles mediated osteoconduction. In the absence of the PRP gel, the bone rested directly on the particle surface. Immediately after implantation, contact with biological fluids induces dissolution of the CaP material, leading to precipitation of the biological apatite. CaP particles, in fact, serve as scaffolding that allows new bone to progress from the periphery to the centre of the implant. PRP can be activated by the addition of thrombin in the presence of calcium chloride and a formed PRP gel. Thrombin activation of PRP causes the release of growth factors, which can improve vascularity and promote tissue regeneration [36, 37]. In addition, use of PRP gel in defects may facilitate release of growth factors by PRP into the immediate local environment and possibly maintain the slow release of these factors [38]. The PRP seemed to modify and promote this standard osteoconduction phenomenon: the newly formed bone almost always grew at a distance from the surface of the granules. The release of growth factors from PRP gel may be highly beneficial for enhancing BMSCs proliferation and differentiation, thus promoting bone regeneration in vivo. However, owing to the limited effect of PRP and low degradability of CaP particles, the effect on bone regeneration was not significantly superior to that of the control group and the BMSCs/PRP gel group.

BMSCs are multipotential cells that play essential roles in bone regeneration. The BMSC sheet has proven effective in bone formation [8] and can effectively preserve cell–cell contact and the extracellular matrix (ECM). Furthermore, the layered cell sheet may mimic the in vivo deposition of bone matrix where osteoblasts are attached on the mineralised sheet [39]. Moreover, the BMSC sheet expresses an osteogenic gene (osteocalcin) and angiogenic gene (VEGF) at high levels and may act as periosteum to promote bone regeneration. Therefore, the BMSC sheet/CaP particles group exhibited more extensive osteogenesis than did the control group, the CaP particles group, or the CaP particles/PRP gel group, which was consistent with a previous report [40] that the effect of BMSCs in bone regeneration was superior to that of PRP. The defects in the BMSC sheet/CaP particles group were regenerated with cortical bone tissue. However, in the control group, BMSCs/PRP gel group, and the CaP particles/PRP gel group, woven bone tissue filled the defects.

Owing to the positive effect of PRP and the BMSC sheet on osteogenesis and angiogenesis, the combination of PRP gel/CaP particles with a BMSC sheet induced the strongest bone regenerative response and greatly accelerated bone regeneration compared with other groups. The BMSC sheet/PRP gel/CaP particles group showed well-distributed and thick cortical bone tissue. This is the first report, to our knowledge, of effective transplantation of a BMSC sheet with PRP gel/CaP particles to promote bone regeneration. A previous study showed that BMSCs in association with PRP lead to more rapid bone regeneration and remodelling [34]. Preliminary clinical results showed that transplantation of BMSCs and PRP was effective during distraction osteogenesis [41].

Vascularisation can enhance bone regeneration by accelerating the differentiation and/or maturation of infiltrating osteoblasts and osteoblast precursor cells during development of new bone. The BMSC sheet/PRP gel/CaP particle group showed obvious osteogenesis, which could be the result of neovascularisation mediated by the BMSC sheet and PRP [42]. PRP is composed of many growth factors known for their proliferative, differentiative and chemo-attractive effects on various cells, which can induce the recruitment of resident stem/progenitor cells or mature cells participating in the development of tissue-engineered bone in vivo. In vitro results showed that PRP could induce the expression of pro-angiogenic factors (VEGF) by BMSCs and the BMSC sheet. These results indicate that PRP induces the secretion of VEGF by BMSCs and BMSC sheet in vitro, which possibly allows the specific recruitment and proliferation of endogenous endothelial cells participating in blood vessel formation in vivo. In addition, the BMSC sheet also highly expressed the VEGF gene. It is possible that the BMSC sheet can self-regulate the secretion of these pro-angiogenic proteins. Therefore, the direct and paracrine effect of the implanted BMSC sheet and the effect of cell adhesion and host cell recruitment of PRP synergistically contributed to vascularization and bone healing [42]. The vascularization facilitates delivery of oxygen and nutrients to the construct and increases the volume of tissue-engineered bone. Furthermore, signals from newly formed vessels may enhance the osteogenic potential of the BMSC sheet, which ultimately influences the maturation of tissue-engineered bone. These results indicate that the BMSC sheet/PRP gel/CaP particles are effective for repair of bone defects.

The effectiveness of any cellular repair approach depends on the retention of cell viability after implantation. In this study, CM-Dil was used to track the transplanted BMSC sheet, and confirmed that the transplanted BMSCs were alive and localised within the defects. Detailed analysis of the tissue sections showed that the red dots were distributed within the defect zone. Therefore, we demonstrated that the implanted BMSCs differentiated into osteoblasts, thereby enhancing the healing of bone defects. However, the contribution of the host cells cannot be excluded. Confocal microscopy showed that not all cells present in the defect were labelled. Therefore, the host cells had already migrated into the defect site and could have contributed to bone regeneration, as was seen in the control group. However, the self-regenerated bone tissue was thinner, woven bone tissue. In addition, a small amount of fibrous tissue filled the surface of the regenerated bone tissue.

The results of the present study indicate that the implanted BMSC sheet and PRP played important roles in promoting bone regeneration. Autologous cell therapy for bone regeneration by combining BMSCs with PRP has many clinical advantages. First, BMSCs can be expanded by cell culture, thus creating a cell sheet. Second, the preparation of the BMSC sheet was simple and required only a culture flask. In clinical cases, a relatively large volume of fresh bone marrow can be harvested or the number of cells expanded by serial passaging. A certain number of such sheets would be able to cover the greater cross-sectional dimensions of human bones. Third, PRP can be easily extracted from autologous blood and activated to form a PRP gel. The procedure is simple and safe with minimal side effects because both BMSCs and PRP are autologous, nontoxic, and nonimmunoreactive. The technique we proposed may be applicable for the repair of bone defects, and could be a useful alternative to allogenic or autologous bone grafts in this regard.

One weakness of the present study was that mechanical tests were not performed. In our study, the bone defects were almost completely regenerated with woven or cortical bone tissue, and the differences between the non-repaired defect area/total cortical bone area among the six groups at four weeks after surgery were not obvious. Derek et al. [43] investigated the effect of different sizes of cortical bone defects on the torsional properties of the distal femur. There was no statistically significant difference in the torque at failure among the 17, 33, and 50 % defects. In a study by Edgerton et al. [44], circular defects that spanned 10–60 % of the bone diameter were created in paired sheep femurs, which were then loaded to failure. The results showed that small defects (10 %) of the bone diameter caused no significant torsional strength reduction. Defects between 20 and 60 % of the bone diameter decreased strength linearly as a function of defect size. Regarding the length of defects, Elias [45] investigated rectangular defects in the posterior cortex of the femur mid-diaphysis using a finite element (FE) model. The results showed that defects with a length of one diameter of bone or shorter had little influence on the femur torsional stiffness or the femur shear-stress distribution. Therefore, the differences in the mechanical test among groups may not be significant. However, if the defects had been segmental bone defects, the mechanical test results would have been more significant. Further studies should involve mechanical testing of segmental bone defects of rats repaired using BMSC sheet/PRP gel/CaP particles.

## Conclusions

CaP particles combined with PRP can synergistically stimulate osteogenic differentiation of BMSCs in osteogenic culture medium. Incorporation of a BMSC sheet along with PRP gel/CaP particles greatly promotes bone regeneration. Such a BMSC sheet and tissue engineering strategies offer therapeutic opportunities to treat bone defects.

## Abbreviations

ALP: alkaline phosphatase
α-MEM: α-minimal essential medium
BMSC: bone marrow-derived mesenchymal stem cell
bp: base pairs
CaP: calcium phosphate
CCK-8: Cell Counting Kit-8
CLSM: confocal laser scanning microscopy
CM-Dil: chloromethyl-benzamidodialkylcarbocyanine
CTAB: hexadecyl (cetyl) trimethyl ammonium bromide
ECM: extracellular matrix
EDS: energy dispersive spectrometer
FBS: foetal bovine serum
IGF: insulin-like growth factor
OCN: osteocalcin
OD: optical density
PBS: phosphate-buffered saline
PCR: polymerase chain reaction
PDGF: platelet-derived growth factor
PRP: platelet-rich plasma
SEM: scanning electron microscopy
TEM: Transmission electron microscopy
TGF: transforming growth factor
VEGF: vascular endothelial growth factor
XRD: x-ray diffraction
